# Toxicity test of flavonoid compounds from the leaves of *Dendrophthoe pentandra* (L.) Miq. using *in vitro* culture cell models

**DOI:** 10.14202/vetworld.2022.2896-2902

**Published:** 2022-12-21

**Authors:** Mochamad Lazuardi, Suharjono Suharjono, Chi-Hsien Chien, Jie-Long He, Chi-Wen Lee, Chia-Kang Peng, Mohammad Sukmanadi, Rahmi Sugihartuti, Lilik Maslachah

**Affiliations:** 1 Sub-division Veterinary-Pharmacy, Faculty of Veterinary Medicine, Universitas Airlangga, Mulyorejo Road, 60115, Surabaya, Indonesia; 2 Department of Pharmaceutics, Faculty of Pharmacy, Universitas Airlangga, Mulyorejo Road, 60115, Surabaya, Indonesia; 3 Department of Veterinary Medicine, Asia University, No. 500, Lioufeng Road, Wufeng District, Taichung 41354, Taiwan

**Keywords:** antimicrobial, Baby Hamster Kidney clone 12 cell culture, cytopathic effect, healthy lifestyle, myeloma cell culture

## Abstract

**Background and Aim:**

The flavonoids from mistletoe are thought to have antimicrobial action. This encouraging finding supports the benefits of medicinal plants as a substitute for synthetic antimicrobials, thus promoting healthy lifestyles. In contrast, it is known that the use of topical drug formulations made from flavonoids of mistletoe (*Dendrophthoe pentandra* (L.) Miq. *Loranthaceae*) with Indonesian name, *Benalu duku* (BD) is required in skin cell irritation. This study aimed to assess the toxic effects of the flavonoid substances of BD, as an initial screening.

**Materials and Methods:**

A myeloma cell line was cultured in Roswell Park Memorial Institute medium, and the Baby Hamster Kidney clone 12 (BHK_21_) cell line was cultured in Dulbecco’s Modified Eagle’s Medium from stock (±9 × 10^7^ cells/mL), and 1.2 mL of culture were distributed into each well of a microtiter plate. Subsequently, 0.2 mL of serially diluted flavonoid compounds (0.5–3 μg/mL) were added to 12 wells for each concentration, as trial groups (including control groups), followed by a 2-day incubation. Observations were performed based on the cytopathic effect (CPE) using an inverted microscope at a magnification of 100×.

**Results:**

Cytopathic effect was detected on the microtiter plate wells for the groups of myeloma and BHK_21_ cells at a flavonoid concentration of 0.5 μg/mL–3 μg/mL.

**Conclusion:**

Flavonoid compounds from BD were safely used for topical treatment of cancer cells at a concentration <2.491 μg/mL, whereas for non-cancerous cells, a concentration <2.582 μg/mL was sufficient (p < 0.05).

## Introduction

The use of medicinal herbs is an alternative approach to avoid exposure to hazardous chemicals, which is also part of the programs aimed at implementing goal 3 of the Sustainable Development Goals 3 pertaining to “Good Health and Well-being” of the United Nations, namely, a healthy lifestyle [1]. The spirit of using medicinal herbs also anticipates problems in the synthetic chemical-based antimicrobial drug sector, namely, the phenomenon of antimicrobial resistance (AMR) [2–4]. This phenomenon can occur due to the minimal stability of synthetic chemical formulations compared with formulations of natural medicinal plants [5, 6]. Changes in molecular structure caused by limited stability will eventually change the microbial receptor as the final target, which, in turn, will lead to the adaptation of the acidic substance of the microbial nucleus as a defensive approach against changes in the chemical structure of drugs. These results in a phenomenon often described as AMR [7–9]. In this regard, the efforts to promote medicinal plant-based formulations will further minimize the AMR phenomenon. Mistletoe, namely, *Benalu duku* (BD), is a parasitic plant that has antiproliferative and androgenic properties and contains bioactive compounds such as alkaloids, flavonoids, polyphenols, saponin, and terpenoids or steroids [10–12]. The bioactive flavonoid compounds of mistletoe include quercetin, an antimicrobial agent. In contrast, flavonoids work as cofactors in plant photosynthesis [13–15]. This process will result in a cycle of energy transfer in all parts of the plant, including the leaves. The production of a thin film that protects the leaves from UV–visible rays is one of the photosynthetic processes. The final product of photosynthesis, when separated using a stratified extraction technique with polar-semi-polar and non-polar solvents, will yield other components dissolved in the extraction solution [16, 17]. The non-quercetin soluble components can be antimicrobial agents, such as the sophoraflavanone G ring and the (−) epigallocatechin ring [18, 19]. Other components are also soluble in extraction solvents, such as aglycones, baicalin, scutellarin, wogonoside, and oroxyloside [20–22]. If a pharmaceutical preparation is based on natural ingredients, the components that are dissolved in the extraction solution will also be formulated in the pharmaceutical preparation that is generated. Thus, the use of components of natural ingredients in the formulation of pharmaceutical preparations was first attempted in topical preparations. Subsequent developments, if deemed sufficiently safe, will be made in oral formulations. The development of oral preparations will be further extended to sterile preparations [23, 24]. Topical formulations must, in principle, ensure that the flavonoids mixed in the formulation are safe for use in eukaryotic cells. This technique can be carried out based on an *in vitro* initial screening model, which will provide an overview of the components of flavonoids that may be toxic to cells. Toxicity screening techniques have been used in the field of veterinary-pharmaceutical science to support the proposal of Russell and Birch in 1959, regarding the last attempt to use experimental animals as a drug test medium [25]. The principle uses the three R’s approach as follows; replacement, reduction, and refinement, so the use of tissue culture is the best option [12].

Based on the background presented above, a screening study was conducted to determine the levels of flavonoids that have a toxic impact on eukaryotic cells. These eukaryotic cells were cultured cell lines that became stabilized after a certain amount of passages [26]. This study aimed to examine the level of toxicity of the polar solvent component of mistletoe extract to eukaryotic cells which was used to obtain flavonoids that would later be used as bioactive drugs. Thus, the toxic components that will be tested in the future can be used as topical drugs. So that using tissue culture techniques, it will reduce the use of experimental animals as a medium for monitoring the toxicity test of a plant compound for medicine.

## Materials and Methods

### Ethical approval

The study did not use experimental animals; therefore, it does not require ethical approval.

### Study period and location

This study was divided into two parts; part 1 of this study was conducted at the Infectious Tropical Diseases Research Centre, Universitas Airlangga, from January 2020 to March 2021, for the preparation of BD leaves as a sample test. Part 2 of this study was performed on the culture medium for cells starting from June 2021 to February 2022 at the Laboratory of Veterinary Pharmacy Science, Faculty of Veterinary Medicine, Universitas Airlangga.

### Materials collection and identification

The leaves of BD were collected from January 2020 to March 2021 from Muara Enim District, Republic of Indonesia at 3° 42’ 41.098” S 104° 0’ 26.046” E. A voucher specimen of BD was identified and deposited in the National Research and Innovation Agency, Directorate of Scientific Collection Management-Republic of Indonesia (Letter No. B-1679/II.6.2/D1.05.07/6/2022). The eukaryotic cell lines used here included the myeloma cell strain P_3_UI, which was cultured in Roswell Park Memorial Institute (RPMI) medium containing 10% fetal bovine serum (FBS), and the Baby Hamster Kidney clone 12 (BHK_21_) cell line, which was cultured in Dulbecco’s Modified Eagle’s Medium (DMEM) containing 10% FBS. The cell lines were obtained from Centre Veterinary Pharmaceutics, Directorate General of Livestock Services, Ministry of Agriculture. Both cell lines were maintained, passaged, and propagated at the Laboratory of Veterinary Pharmacy Science, Faculty of Veterinary Medicine, Universitas Airlangga. The flavonoid separation process was carried out at the Tropical Research Diseases Universitas Airlangga through semi-preparation of high-performance liquid chromatography using ultra violet-photo diode array detector equipped with a Shimadzu LC-6A AP pump, a DGU-20A5 degasser, a modul-20A communication cable, and a PDA detector type SPD-M20A completely with an FRC-10 A fraction collector. Microtiter 36-well flat-bottom polystyrene plates were used at a capacity of 1.4 mL in sterile conditions.

### Research design

The research design consisted in a true experimental model by performing a post-test only in the control groups at the sample size presented in Table-1. The table was used to calculate the number of microplates well tests at α and β 0.05, assuming that the flavonoid toxicity test in cell culture was 100% accurate with a 70% representative population [27]. Twelve microplate wells were used for each concentration. Furthermore, since we used six serially diluted concentrations with five replications, the total number of wells used was 360. The control groups were designed as follows: One group for the myeloma cell line and one group for the BHK_21_ cell line. The total number of wells used for the control groups was 24. Briefly, all samples were seeded (N) into 384 wells of 11 microtiter plates. The toxicity assessment was performed based on the cytopathogenic effect (CPE) on cell growth. The criteria used to assess the CPE phenomenon are presented in Table-2 [13, 14, 28, 29].

**Table-1:** Samples used for the testing of flavonoid toxicity in eukaryote cells.

Accuracy of the flavonoid toxicity test in cell culture (%)										Representative population (%)
10	20	30	40	50	60	70	80	90	100	0
53	28	17	12	9	7					10
	270	83	42	26	18	13	9	7		20
		402	111	53	31	20	14	9		30
			294	128	58	32	20	13		40
				539	134	58	31	18	7	50
					539	128	53	26	9	60
						494	111	42	12	70
							402	83	17	80
								270	26	90
									53	100

**Table-2:** Criteria used to assess the CPE in the wells of the microtiter plates.

Criteria CPE	Observation on an inverted microscope
True CPE	In one field of view, a single movement of the ocular lens to delineate the letter S detected a hollow cell culture arrangement and floating cells on the surface of the medium [13, 14]. If more than 1 cavity was found during the S-shaped movement, it was determined that the wells exhibited CPE [28, 29].

CPE=Cytopathic effect

### Flavonoid separation

The leaves of BD were pulverized and weighed carefully, extracted with the maceration method using methanol, and then concentrated using a rotary evaporator machine. The crude extract of BD leaves was partitioned using aqua pro-chromatographic and ethyl acetate for obtained crude extract free from tannin. To obtain total flavonoid fraction, the concentrated extract was partitioned with methanol and n-hexane [30]. For the flavonoid purification process, a gradient elution system was used of 3 s each on a preparative column SUPELCOSIL LC-PAH with a 5 μm particle size, an L × ID of 25 cm × 4.60 mm, and water/methanol ratios of 9:1, 8:2, 7:3, and 6:4 as the mobile phase eluent. The HPLC grade of quercetin from Sigma at catalog no. Q4951 was used as an analog standard of flavonoid [31]. The purified products from separate processes were placed in storage bottles and dried on a rotary evaporator to a powder. The purified flavonoids were determined qualitatively using a 5% of FeCl_3_ solution as a color indicator in the black solution. Other tests of flavonoid content in the concentrated extract of BD included the use of the Shinoda reagent, Mg, and HCl, which yielded a new color, as an indicator of the presence of these compounds. Flavonoids in the dried form were assessed by adding phosphate-buffered saline (PBS) at pH 6.80, to prepare 10 μg/mL of a stock solution (w/v). The stock of flavonoid compounds was serially diluted using PBS as follows: 0.5, 1, 1.5, 2, 2.5, and 3 μg/mL. All serial dilutions were filtered (Ø = 0.20 μm) and kept in colored glass bottles until use.

### Stock cell preparation

Myeloma and BHK_21_ cell lines from a stock kept in a deep freezer (−80°C) were thawed and revived to propagate each cell line in 100 mL Roux bottles using at least 9 × 10^8^ cells/mL as follows: The stocked cells were rinsed with PBS twice and centrifuged at 1000× *g* (30 min at 20°C). Subsequently, 50 mL of medium (RPMI for myeloma cells and DMEM for BHK_21_ cells) containing 10% FBS was added to the supernatant, followed by vigorous shaking for 5 min and distribution of 10 mL of each cell culture into Roux bottles. Finally, the bottle was closed with a rubber stopper that was kept loose and incubated in a CO_2_ incubator for 2 days at 37°C.

### Toxicity test protocol

The myeloma and BHK_21_ cells cultured in the Roux bottles were harvested as follows: (a) The old medium was discarded; (b) 3 mL of a 1% trypsin solution was added and the bottles were shaken well for 3 min; (c) 10 mL of medium containing FBS was added (RPMI for myeloma cells and DMEM for BHK_21_ cells); (d) 10 mL of each cell was placed in tubes; (e) 1.2 mL of the cells was distributed onto the wells of a microplate (72 wells for trial groups and 24 wells for control groups); (f) serial dilutions of flavonoids were added (0.2 mL, or approximately 17% of the volume of the well (each concentration of flavonoid was represented in 12 wells of the microtiter plates); (g) 0.2 mL of medium (RPMI for myeloma cells and DMEM for BHK_21_ cells) was added to the 24 control wells; (h) all microplates were incubated for 2 days; and (i) observations were carried out on 3 days post-incubation using an inverted microscope at 100× (10× objective lens with 10× ocular lens), for the assessment of the CPE phenomenon in each well compared with the control wells.

### Statistical analysis

The results of the observation of the wells corresponding to myeloma and BHK_21_ cells at concentrations of 0.5–3 mg/mL as the trial groups were assessed by probit analysis, to determine the endpoint percentages. The characterized toxicity in both myeloma and BHK_21_ cells was analyzed in the groups starting at CPE cases, followed by one-way analysis of variance for the the analysis of significance at 5% using Statistical Package for the Social Science 24.0 (IBM SPSS Software, Chicago, Il, USA).

## Results

The pulverization of 1 kg of BD leaves yielded 400 mg of pure flavonoids, which were then determined qualitatively through the addition of 5% FeCl_3_ to obtain a black-colored solution, as well as through other reactions using the Shinoda reagent, Mg, and HCl, to obtain a reddish color solution. The results of the toxicity tests of flavonoids at serial dilutions of 0.5, 1, 1.5, 2, 2.5, and 3 μg/mL, with five replicates, against myeloma and BHK_21_ cell lines are presented in Table-3.

**Table-3:** Results of the toxicity test of flavonoids extracted from the leaves of *Dendrophthoe pentandra* L. (Miq.) using a myeloma cell line (P_3_UI) and the BHK_21_ cell line based on the CPE.

Flavonoid (μg/mL)	Replicate	Wells	Number of wells exhibiting CPE		p (2.5–3 μg/mL)

			Myeloma cell culture	BHK_21_ cell culture	
0.0	0	12	0	0	0.012
0.5	1	12	0	0	
	2	12	0	0	
	3	12	0	0	
	4	12	0	0	
	5	12	0	0	
1	1	12	0	0	
	2	12	0	0	
	3	12	0	0	
	4	12	0	0	
	5	12	0	0	
1.5	1	12	0	0	
	2	12	0	0	
	3	12	0	0	
	4	12	0	0	
	5	12	0	0	
2	1	12	0	0	
	2	12	0	0	
	3	12	0	0	
	4	12	0	0	
	5	12	0	0	
2.5	1	12	0	0	
	2	12	1	0	
	3	12	1	1	
	4	12	1	0	
	5	12	1	0	
3	1	12	1	0	
	2	12	2	0	
	3	12	2	1	
	4	12	1	1	
	5	12	1	1	

CPE=Cytopathic effect

The endpoint percentages of flavonoid concentrations that yielded a CPE in the myeloma and BHK_21_ cell cultures were started at 2% with concentrations at 2.066 μg/mL and 2.360 μg/mL, respectively. The descriptions provided in Table-3 indicated that the percentage of wells of the microtiter plate that exhibited the CPE condition in the myeloma cell culture groups after contact with the BD flavonoid extract at 2.5 μg/mL was 6.6%, whereas that observed in the BHK_21_ cell culture groups was 1.6%. The concentration of 3 μg/mL of flavonoids in the BD extract triggered a CPE in a mean of 11.7% of the wells in the myeloma cell culture groups, whereas it triggered a CPE condition in 5% of the wells in the BH_21_ cell culture groups.

**Figure-1:**
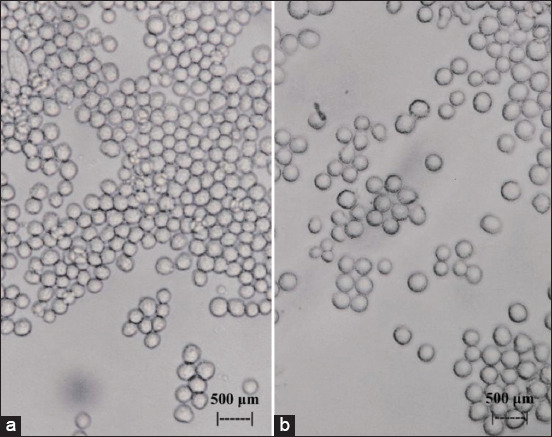
Myeloma cultured cells after exposure to 2.5 μg/mL of flavonoids from the leaves of *Dendrophthoe pentandra* L. Miq. (a) and myeloma cultured cells after exposure to 3 μg/mL of flavonoids extracted from the leaves of *Dendrophthoe pentandra* L. Miq. (b) were examined using an inverted microscope at 100×.

**Figure-2:**
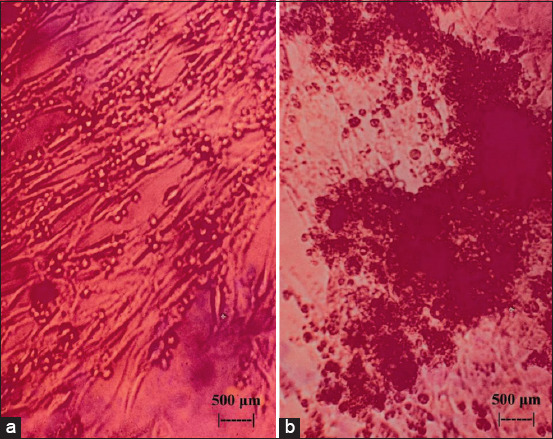
Baby Hamster Kidney clone 12 (BHK_21_) cultured cells after exposure to 2.500 μg/mL of flavonoids extracted from the leaves of *Dendrophthoe pentandra* L. Miq. (a) and BHK_21_ cultured cells after exposure to 3 μg/mL of flavonoids extracted from the leaves of *Dendrophthoe pentandra* L. Miq. (b) were examined using an inverted microscope at 100×.

**Figure-3:**
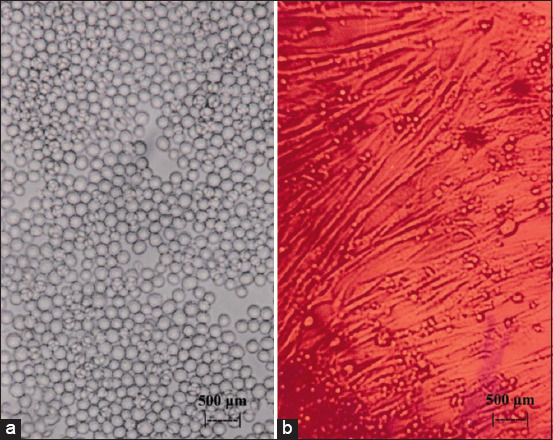
Myeloma cells cultured in Roswell Park Memorial Institute medium, as a control group (a) and BHK_21_ cells cultured in Dulbecco’s Modified Eagle’s Medium, as a control group (b), were assessed using an inverted microscope at 100×.

Figure-1 shows the initial incidence of CPE in myeloma cell culture after the administration of flavonoids extracted from the leaves of BD; at concentrations of 2.5 and 3 μg/mL, all myeloma cell cultures exhibited CPE. In turn, Figure-2 shows the onset of CPE in BHK_21_ cell culture after the administration of flavonoids extracted from the leaves of BD (*Dendrophthoe pentandra* L. Miq.); at concentrations of (a) 2.5 and (b) 3 μg/mL, it was also found that all BHK_21_ cell cultures exhibited CPE (b). Figure-3 shows myeloma (a) and BHK_21_ (b) cell cultures without the CPE condition, which was detected in the control group.

## Discussion

An analysis of the data pertaining to the microplate wells with exposure to flavonoids at 2.500 μg/mL among myeloma cells showed that the mean percentage of wells that experienced CPE was low (<20%). The CPE criteria of <20% in toxicity testing in cell tissue culture have been mentioned in the previous research reports [32, 33]. These results indicate that, in formulations containing flavonoids, their concentration should not exceed 2.500 μg/mL (w/v). Moreover, this concentration was still below the 10% endpoint of proliferating eukaryotic cells, which were represented here by the myeloma cell culture groups (2.530 μg/mL). In turn, exposure of non-proliferate eukaryotic cells, which were represented here by BHK_21_ cells, yielded a relatively lower percentage of microplate wells that experienced CPE (1.6%). When the flavonoid concentration was increased to 3 μg/mL, the number of microplate wells that experienced CPE was <20% for both cell types (myeloma cell culture groups, 11.7%, and BHK_21_ cell culture groups, 5%). Thus, exposure to flavonoids at a concentration of up to 3 μg/mL is still considered not to have a harmful impact on eukaryotic cells, considering that the CPE generated was below 20%.

To date, the toxic compounds in the flavonoid extract of BD leaves remain mostly unknown. Based on the literature, the compounds of other plants that caused toxic effects in eukaryotic cells were as follows: Hydroxycinnamic acids, proanthocyanidin dimers, compounds of polyphenols, flavones, 5-feruloylquinic acid, methoxycinnamic acids, and hydroxybenzoic acids [34, 35]. When combined with buffer elements or reacted with salt-forming elements, these compounds become neutral, thus not having toxic effects. The technique of administering this combination of compounds has been widely developed by the cosmetic industry for the formulation of semi-synthetic topical pharmaceutical preparations. Such development will be beneficial while also producing new compounds resulting from the combination of natural and synthetic compounds.

A sensitivity assessment to the exposure of flavonoid BD using the indicator CPE to culture cells between myeloma cells and BHK_21_ cells showed that myeloma was more sensitive than BHK_21_. Figure-1 shows cultured myeloma cells exposed to flavonoids at 2.5 μg/mL (a), which exhibited a relatively large number of holes, whereas exposure to three flavonoids at μg/mL (b) slowed down cell growth further. This was in contrast with the results shown in Figure-3, where cultured myeloma cells were not exposed to flavonoids (a). This is because myeloma cells have a higher metabolism than do BHK_21_ cells, considering that myeloma is a type of cancer cell [36, 37]. An indication of the high metabolism of myeloma cells was observed in the difference in the color of the medium, as the medium of myeloma cells turned pale faster than that of BHK_21_ cells (Figures-1 and 2). This is because cells in culture require more energy for growth, especially among cancer cells [38, 39]. Thus, exposure to flavonoids in small amounts will disrupt the metabolic system of cells in culture. This was different for BHK_21_ cells, which calculated by probit analysis were relatively resistant to flavonoid exposure even at a concentration of 2.484 μg/mL, as only 3% of the wells exhibited CPE among the five replicates (Figure-2, 5% CPE of 3 μg/mL). In Figure-3, the BHK_21_ control cells were relatively fully grown in the microplate well (A). The resistance of BHK_21_ cultured cells to exposure to BD flavonoids is attributed to the fact that their metabolic system is not excessive so that other energy sources can be used to defend itself against toxic elements that interfere with the sulfur metabolism. One of the energy sources that are used for self-defense is the thickening of the lipid bilayer of BHK_21_ cells while keeping the toxic compounds from flavonoids from binding to elements from the cell cytoplasm. This pathway was carried out through the high lipophilicity of the surface of BHK_21_ cell culture. Entry of flavonoid toxic elements into the cytoplasm can occur when the lipid bilayer pores on the cell surface are bound, thus allowing flow into the cytoplasm through a phagocytosis mechanism [40]. This phenomenon triggers a change in the acid-base balance in the cytoplasm of the cell, which, in turn, affects the metabolic system of cellular respiration, eventually leading to cell death. Figure-2 shows that the dead cultured cells float to the top (b), which was not observed for the control cells, as shown in Figure-3b.

The use of BD flavonoids for topical preparations or cosmetics in pharmaceutical applications must consider the toxic factors identified in *in vitro* studies. Thus, the strategy that is widely used is to apply a concentration (μg/mL) of flavonoid components that do not pose a risk of developing toxic cell phenomena. Thus, a flavonoid concentration below 2.066 μg/mL is most suitable for the topical preparation of cancer eukaryotic cells. An increase in concentration up to the 9% endpoint (2.491 μg/mL) for proliferate eukaryotic cells, such as myeloma, still does not pose a major toxic risk. In the *in vitro* study using BHK_21_ cells, a concentration of 2.916 μg/mL caused CPE at 9%. If the flavonoid level is increased to a level slightly below the 10% endpoint of BHK_21_ cultured cells (below 2.968 μg/mL), the risk of toxicity remains relatively low.

Toxicity test studies of drug formulation compounds, including flavonoids, using the tissue culture method are instrumental, especially for the initial screening of a new formula. This method can be used for deeper testing by employing tools such as experimental animals. Thus, this method can help assess the part to be tested using experimental animals. The weakness of the toxicity test method using tissue culture is that a specific cell type is needed to represent the skin or organ. This is not easy, considering that preparing tissue cultures from specific organs to generate cell lines are a time-consuming process. In this study, antibiotics and antifungals were not added to the media used because these agents may interact pharmaceutically with flavonoids.

## Conclusion

This study concluded that the flavonoids extracted from BD leaves can be formulated for pharmaceutics of topical treatment for cancer cells at a concentration below 2.491 μg/mL. In comparison, for non-cancerous cells, it can be used at levels below 2.582 μg/mL. Finally, the toxicity test technique for pharmaceutical preparations can be carried out using the same method if the cultured cells have developed into cell lines.

## Authors’ Contributions

ML: Conceived and designed the experiments. ML: Performed the experiments. ML, CC, and JH: Analyzed and interpreted the data. ML: Statistical analysis. ML: Contributed reagents, materials, analysis tools, or data. ML, CC, JH, CL, CP, SS, RS, MS, and LM: Wrote the paper. All authors have read and approved the final manuscript.

## References

[1] Kumar S, Kumar N, Vivekadhish S (2016). Millennium development goals (MDGs) to sustainable development goals (SDGs):Addressing unfinished agenda and strengthening sustainable development and partnership. Indian. J. Community Med.

[2] Vaou N, Stavropoulou E, Voidarou C, Tsigalou C, Bezirtzoglou E (2021). Towards advances in medicinal plant antimicrobial activity:A review study on challenges and future perspectives. Microorganisms.

[3] Radi F.Z, Bouhrim M, Mechchate H, Al-Zahrani M, Qurtam A.A, Aleissa A.M, Drioiche A, Handaq N, Zair T (2021). Phytochemical analysis, antimicrobial and antioxidant properties of *Thymus zygis* L. and *Thymus willdenowii* boiss essential oils. Plants (Basel).

[4] Ralte L, Khiangte L, Thangjam N.M, Kumar A, Singh Y.T (2022). GC-MS and molecular docking analyses of phytochemicals from the underutilized plant, *Parkia timoriana* revealed candidate anti-cancerous and anti-inflammatory agents. Sci. Rep.

[5] Prestinaci F, Pezzotti P, Pantosti A (2015). Antimicrobial resistance:A global multifaceted phenomenon. Pathog. Glob. Health.

[6] Najmi A, Javed S.A, Al Bratty M, Alhazmi H.A (2022). Modern approaches in the discovery and development of plant-based natural products and their analogues as potential therapeutic agents. Molecules.

[7] Chhetri G, Kim I, Kim J, So Y, Seo T (2022). *Chryseobacterium tagetis* spp. nov., a plant growth-promoting bacterium with an antimicrobial activity isolated from the roots of a medicinal plant (*Tagetes patula*). J.Antibiot (Tokyo).

[8] Demachi A, Ohte S, Uchida R, Shin-ya K, Ohshiro T, Tomoda H, Ikeda H (2022). Discovery of prescopranone, a key intermediate in scopranone biosynthesis. J. Antibiot(Tokyo).

[9] Halimehjani A.Z, Dehghan F, Tafakori V, Amini E, Hooshmand S.E, Nosood Y.L (2022). Synthesis of novel antibacterial and antifungal dithiocarbamate-containing piperazine derivatives via re-engineering multicomponent approach. Heliyon.

[10] Tungmunnithum D, Thongboonyou A, Pholboon A, Yangsabai A (2018). Flavonoids and other phenolic compounds from medicinal plants for pharmaceutical and medical aspects:An overview. Medicines (Basel).

[11] Singh P, Arif Y, Bajguz A, Hayat S (2021). The role of quercetin in plants. Plant. Physiol. Biochem.

[12] Long Y, Yang Y, Pan G, Shen Y (2022). New insights into tissue culture plant-regeneration mechanisms. Front. Plant. Sci.

[13] Górniak I, Bartoszewski R, Króliczewski J (2019). Comprehensive review of antimicrobial activities of plant flavonoids. Phytochem. Rev.

[14] Ni Y.W, Lin K.H, Chen K.H, Wu C.W, Chang Y.S (2020). Flavonoid compounds and photosynthesis in *Passiflora* plant leaves under varying light intensities. Plants (Basel).

[15] González A, Casado J, Lanas Á (2021). Fighting the antibiotic crisis:Flavonoids as promising antibacterial drugs against *Helicobacter pylori* infection. Front. Cell. Infect Microbiol.

[16] Bhandari S, Khadayat K, Poudel S, Shrestha S, Shrestha R, Devkota P, Khanal S.,, Marasini B.P (2021). Phytochemical analysis of medicinal plants of Nepal and their antibacterial and antibiofilm activities against uropathogenic *Escherichia*
*coli*. BMC Complement. Med. Ther.

[17] Manso T, Lores M, de Miguel T (2021). Antimicrobial activity of polyphenols and natural polyphenolic extracts on clinical isolates. Antibiotics (Basel).

[18] Park S, Kim J, Shin YK, Kim K.Y (2021). Antimicrobial activity of 4-hydroxyderricin, sophoraflavanone G, acetylshikonin, and kurarinone against the bee pathogenic bacteria *Paenibacillus*
*larvae* and *Melissococcus*
*plutonius*. J. Apic. Res.

[19] Zhao W, Liu Z, Liang X, Wang S, Ding J, Li Z, Wang L, Jiang Y (2022). Preparation and characterization of epigallocatechin-3-gallate loaded melanin nanocomposite (EGCG @MNPs) for improved thermal stability, antioxidant and antibacterial activity. LWT.

[20] Hikmawanti N.P.E, Ramadon D, Jantan I, Mun'im A (2021). Natural deep eutectic solvents (NADES):Phytochemical extraction performance enhancer for pharmaceutical and nutraceutical product development. Plants(Basel).

[21] Askey B.C, Liu D, Rubin G.M, Kunik A.R, Song Y.H, Ding Y, Kim J (2021). Metabolite profiling reveals organ-specific flavone accumulation in *Scutellaria* and identifies a scutellarin isomer isoscutellarein 8-*O*-b-glucuronopyranoside. Plant. Direct.

[22] Oomen W.W, Begines P, Mustafa N.R, Wilson E.G, Verpoorte R, Choi Y.H (2020). Natural deep eutectic solvent extraction of flavonoids of *Scutellaria baicalensis* as a replacement for conventional organic solvents. Molecules.

[23] Watson C.J, Whitledge J.D, Siani A.M, Burns M.M (2021). Pharmaceutical compounding:A history, regulatory overview, and systematic review of compounding errors. J. Med. Toxicol.

[24] Pandey A, Jatana G.K, Sonthalia S (2022). Cosmeceuticals.

[25] Fishcher I, Milton C, Wallace H (2020). Toxicity testing is evolving!*Toxicol*. Res(Camb).

[26] Christodoulou I, Gouliemaki M, Kritios A, Zoumpourlis P, Koliakos G, Zoumpourlis V (2022). Suitability of human mesenchymal stem cells derived from fetal umbilical cord (Wharton's jelly) as an alternative *in vitro* model for acute drug toxicity screening. Cells.

[27] Stephens L, Hintz-Prunty W, Bengtsson H.I, Proudfoot JA, Patel S.P, Broome H.E (2017). Impact of integrating rumke statistics to assist with choosing between automated hematology analyzer differentials vs manual differentials. J. Appl. Lab. Med.

[28] Kamiloglu S, Sari G, Ozda T, Capanoglu E (2020). Guidelines for cell viability assays. Food. Front.

[29] Kitaeva K.V, Rutland C.S, Rizvanov A.A, Solovyeva V.V (2020). Cell culture based *in vitro* test systems for anticancer drug screening. Front. Bioeng. Biotechnol.

[30] Hardiyanti R, Marpaung L, Adnyana I.K, Simanjuntak P (2019). Biochemical evaluation of duku's mistletoe leave (*Dendrophthoe pentandra (L.) Miq)* extract with antidiabetic potential. Rasayan J. Chem.

[31] Mochamad L, Hermanto B, Hestianah E.P (2019). Determination of progesterone compounds in the crude methanol extract of *Benalu duku* leaves. Vet. World.

[32] Obi R.K, Chikwendu C.I, Shenge J.A (2020). *In vitro* cytotoxic effects of extracts of fourteen medicinal plants of Nigerian origin on vero cells. Adv. J. Toxicol. Curr. Res.

[33] Hirose R, Watanabe N, Bandou R, Yoshida T, Daidoji T, Naito Y, Itoh Y.,, Nakaya T (2021). A cytopathic effect-based tissue culture method for HCoV-OC43 titration using TMPRSS2-expressing veroE6 cells. mSphere.

[34] Sayago-Ayerdi S, García-Martínez D.L, Ramírez-Castillo A.C, Ramírez-Concepción H.R.,, Viuda-Martos M (2021). Tropical fruits and their Co-products as bioactive compounds and their health effects:A review. Foods.

[35] Insanu M, Karimah H, Pramastya H, Fidrianny I (2021). Phytochemical compounds and pharmacological activities of *Vitis vinifera* L.:An updated review. Biointerface. Res. Appl. Chem.

[36] Cohen K, Abadi U, Hercbergs A, Davis P.J, Ellis M, Ashur-Fabian O (2018). The induction of myeloma cell death DNA damage by tetrac, a thyroid hormone derivative. Endocr. Relat. Cancer.

[37] Sartori R, Leme J, Caricati C.P, Tonso A, Núñez E.G.F (2018). Model comparison to described BHK-21cell growth and metabolism in stirred tank bioreactors operated in batch mode. Braz. J. Chem. Eng.

[38] Santana-Codina N, Mancias J.D, Kimmelman A.C (2017). The role of autophagy in cancer. Annu Rev Cancer Biol.

[39] Verma A, Verma M, Singh A (2020). Animal tissue culture principles and applications. Anim. Biotechnol.

[40] Kerimi A, Williamson G (2018). Differential impact of flavonoids on redox modulation, bioenergetics, and cell signaling in normal and tumor cells:A comprehensive review. Antioxid. Redox Signal.

